# Author Correction: Lunar primitive mantle olivine returned by Chang’e-6

**DOI:** 10.1038/s41467-025-60198-2

**Published:** 2025-05-22

**Authors:** Si-Zhang Sheng, Shui-Jiong Wang, Qiu-Li Li, Shitou Wu, Hao Wang, Jun-Xiang Hua, Zhenyu Chen, Jin-Hua Hao, Bo Zhang, Yongsheng He, Jian-Ming Zhu

**Affiliations:** 1 State Key Laboratory of Geological Processes and Mineral Resources, China University of Geosciences (Beijing), Beijing, China; 2 Frontiers Science Center for Deep-time Digital Earth, China University of Geosciences (Beijing), Beijing, China; 3 State Key Laboratory of Lithospheric and Environmental Coevolution, Institute of Geology and Geophysics, Chinese Academy of Sciences, Beijing, China; 4 MNR Key Laboratory of Metallogeny and Mineral Assessment, Institute of Mineral Resource, Chinese Academy of Geological Sciences, Beijing, China; 5 Institute of Earth Sciences, China University of Geosciences (Beijing), Beijing, China; 6 The Key Laboratory of Orogenic Belts and Crustal Evolution, School of Earth and Space Sciences, Peking University, Beijing, China

**Keywords:** Geochemistry, Mineralogy

Correction to: *Nature Communications* 10.1038/s41467-025-58820-4, published online 23 April 2025

In the version of the article initially published, two references were missing from the “In situ trace element analysis” section of the Methods: Batanova, V. G., et al. New olivine reference material for in situ microanalysis. *Geostand. Geoanal. Res*. **43**, 453–473 (2019) and Jochum, K. P. et al. MPI-DING reference glasses for in situ microanalysis: new reference values for element concentrations and isotope ratios. *Geochem. Geophys. Geosyst*. 10.1029/2005GC001060 (2006). These have now been added as refs. 54 and 55 in the HTML and PDF versions of the article.

