# Supplementary material for: Lunar primitive mantle olivine returned by Chang’e-6
Source: Nat Commun. 2025 Apr 23;16:3759. doi: 10.1038/s41467-025-58820-4 (PMC12019214; doi:10.1038/s41467-025-58820-4)
Supplement: Supplementary file 1 — Supplementary Information [file 41467_2025_58820_MOESM1_ESM.pdf]

Supplementary Information for

Lunar primitive mantle olivine returned by Chang'e-6

Si-Zhang Sheng[1], Shui-Jiong Wang[1,2]*, Qiu-Li Li[3], Shitou Wu[3], Hao Wang[3], Jun-Xiang Hua[1], Zhenyu Chen[4], Jin-Hua Hao[5], Bo-Zhang[6], Yongsheng He[1,2], Jian-Ming Zhu[1,2]

[1] State Key Laboratory of Geological Processes and Mineral Resources, China University of Geosciences (Beijing); Beijing, 100083, China.

[2] Frontiers Science Center for Deep-time Digital Earth, China University of Geosciences (Beijing); Beijing, 100083, China.

[3] State Key Laboratory of Lithospheric and Environmental Coevolution, Institute of Geology and Geophysics, Chinese Academy of Sciences, Beijing 100029, China.

[4] MNR Key Laboratory of Metallogeny and Mineral Assessment, Institute of Mineral Resource CAGS, Beijing 100037, China

[5] Institute of Earth Sciences, China University of Geosciences, Beijing 100083, China.

[6] The Key Laboratory of Orogenic Belts and Crustal Evolution, School of Earth and Space Sciences, Peking University; Beijing,100871, China.

*Corresponding author. wsj@cugb.edu.cn

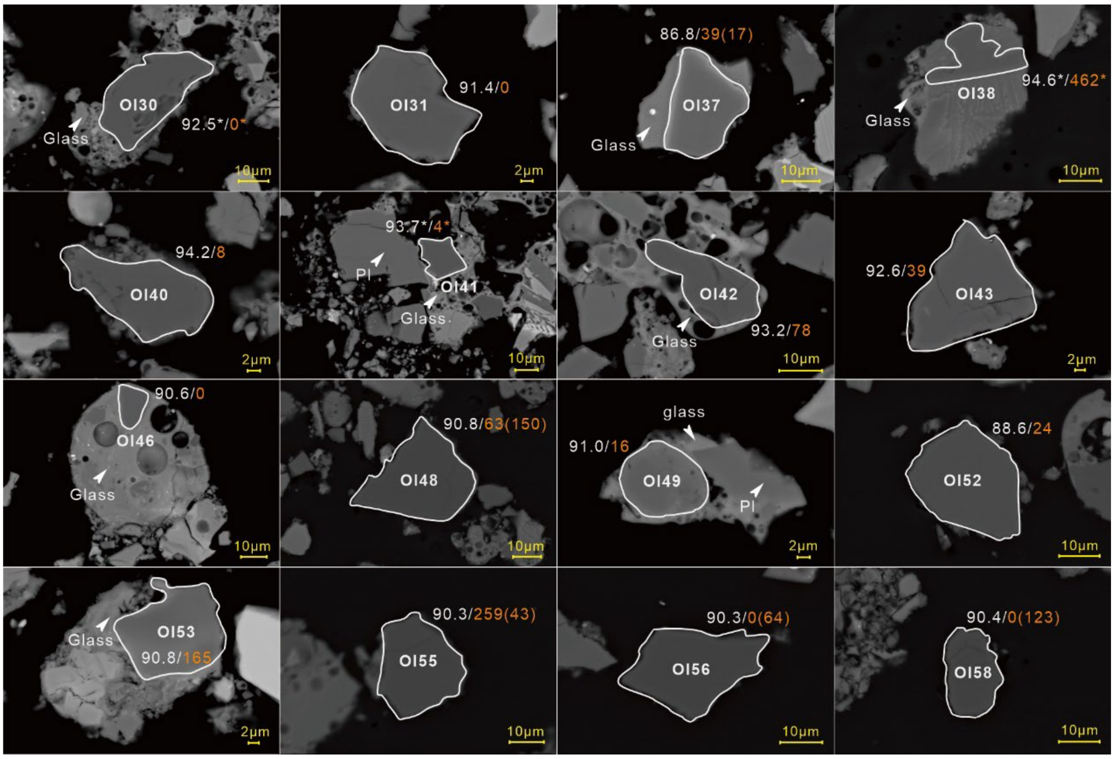

Supplementary Fig. 1. Representative backscattered electron (BSE) images of the CE-6 olivine fragments. Pl, plagioclase. Numbers are the Fo values (white) and Ni contents (orange) of olivines (* stands for average values from multiple analyses on a single olivine grain). LA-ICPMS data are shown in parentheses if analyzed.

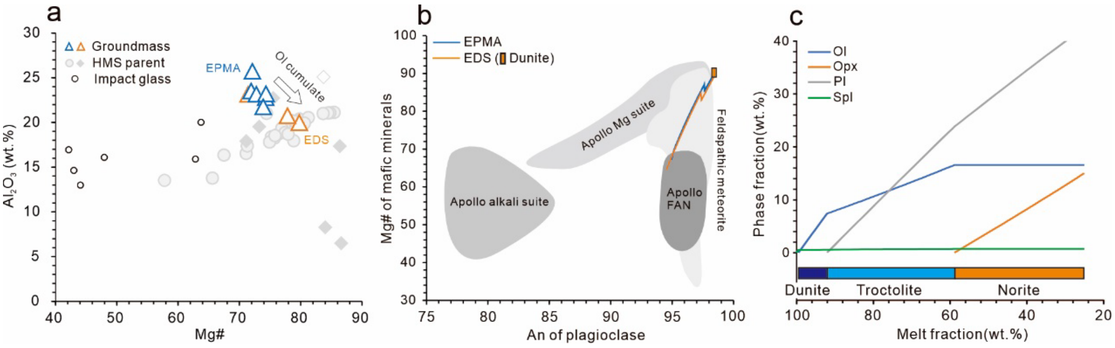

Supplementary Fig. 2. Geochemistry of the groundmass. (a) Compositions of the troctolitic groundmass determined by electron microprobe (EPMA, blue triangles) and energy dispersive spectroscopy (EDS, orange triangles) analyses compared to the parental liquid of lunar high Mg suite (HMS) by experiments [1](solid circles) and modeling[2-5] (diamonds). Also shown are compositions of impact glasses adjacent to single olivine fragments in the CE-6 soils (unfilled circles). **(b)** An-Mg# plot showing the crystal fractionation of the average composition of groundmass by EPMA and EDS at 1 bar. The composition of dunite, produced before plagioclase crystallized in the case of groundmass by EDS is illustrated as an orange rectangle. Compositional fields for the Apollo samples and feldspathic meteorites are from ref. 6 and ref. 7 (after ref. 8) respectively. **(c)** Cumulate mineral proportions as a function of residual melt fraction produced by the crystal fractionation of the groundmass at 1bar. Rock types identified by mineral assemblages are shown beneath. Ol, olivine; Opx, orthopyroxene; Pl, plagioclase; Spl, spinel.

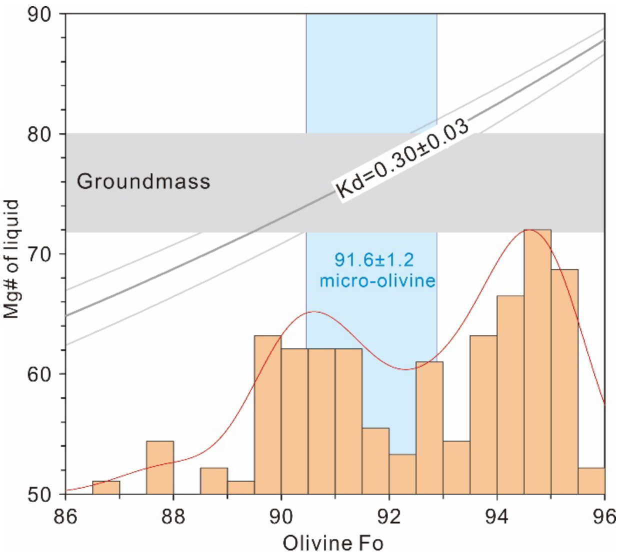

Supplementary Fig. 3. Calculated Mg# of liquid equilibrated with olivine grains in this study, using Kd= 0.30 ± 0.03 [9]. Fo content of olivine is shown as a histogram with a Kernel Smooth curve. Also shown are the compositional field of the groundmass (grey) and average composition of the micro-olivine (blue) in this study.

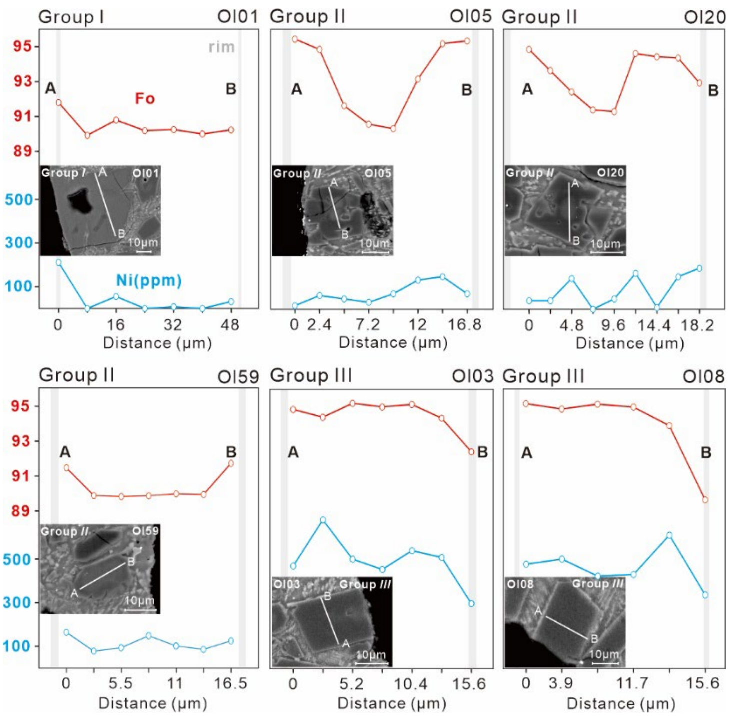

Supplementary Fig. 4. Detailed Electron probe traverses across different groups of olivine crystals in the lithic fragment. Note that the rim of olivine grain is too narrow for actual analyses (grey bars).

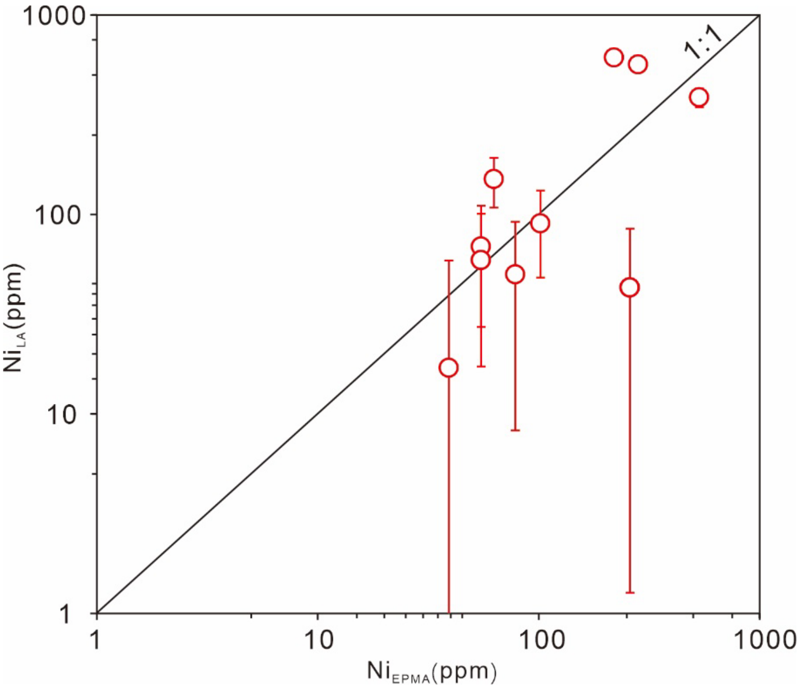

Supplementary Fig. 5. Comparison of Ni contents of CE-6 olivines analyzed by EPMA and LA-ICPMS. Error bars represent 2se of LA-ICPMS analysis.

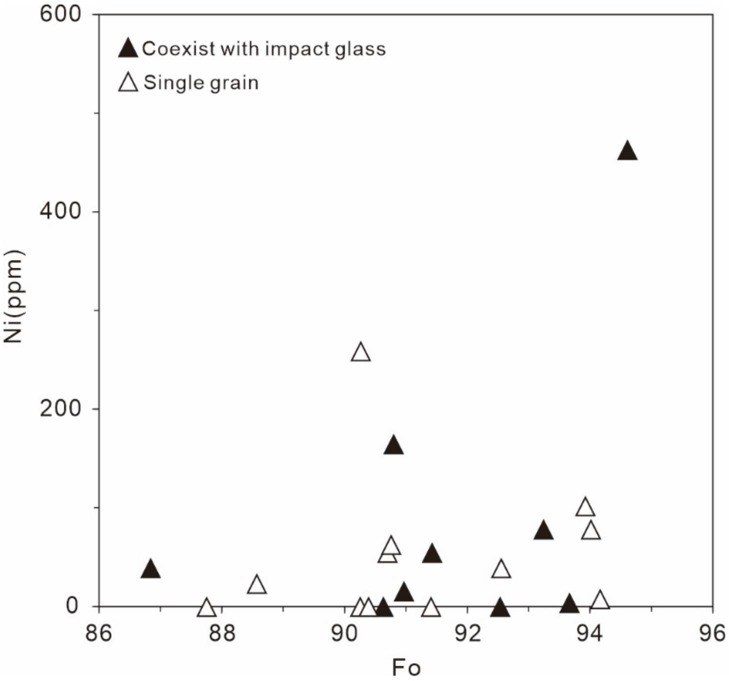

Supplementary Fig. 6. Fo versus Ni content of the single olivine fragments coexist with the impact glass (filled) compared with those do not (unfilled).

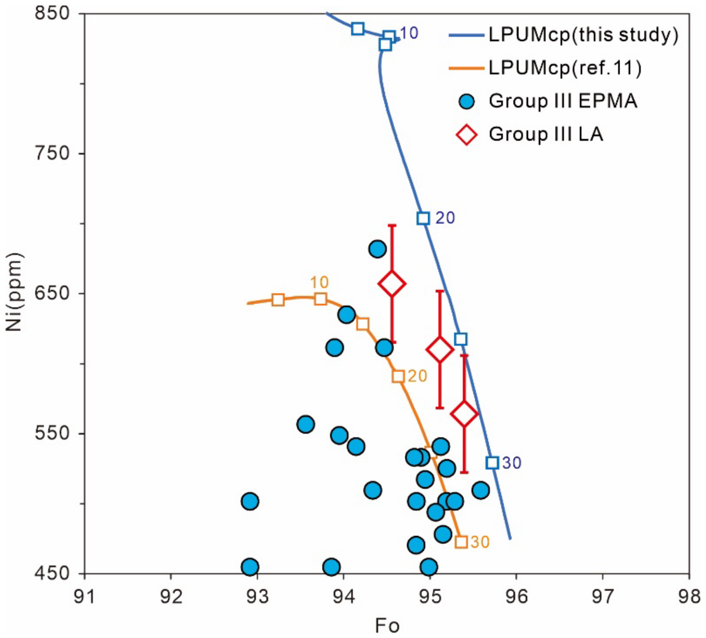

Supplementary Fig. 7. Thermodynamic modeling of Fo versus Ni content of olivine during equilibrium melting of the LMO cumulate pile (LPUMcp) at 50 PCS of the LMO with an initial composition of the LPUM[10] from this study (blue line) and ref. 11 (orange line). The squares stand for the melting degree (%). The EPMA/LA-ICPMS analyses of Group III olivines are shown as circles/ diamonds. Error bars represent 2se of LA-ICPMS analysis.

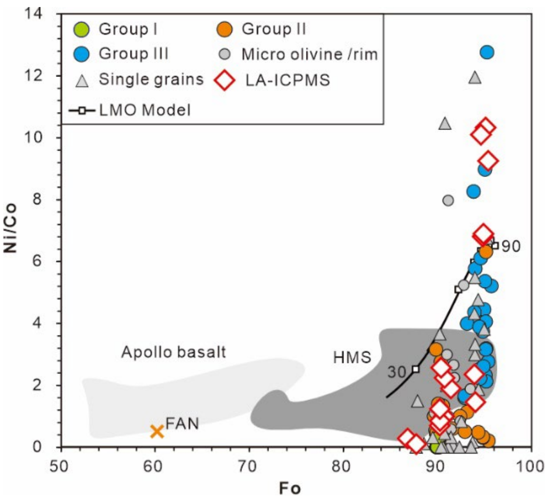

Supplementary Fig. 8. Ni/Co ratios versus Fo values of the CE-6 olivines. Olivines in lunar basalts[12], ferroan anorthosite[13] (FAN, cross) and Apollo Mg-suite rocks[13](HMS) are also shown for comparisons. The curves are modeled compositions of olivines crystallized from the LMO with an initial composition of the LPUM[10]. EPMA analyses of olivine grains in the particular lithic fragment are shown in circles and those of single olivine grains are shown in triangles. LA-ICPMS data are shown in diamonds. Modeled compositions of olivine crystallized from the LMO with an initial depth of 1000 km is shown in the solid line. The squares stand for the proportion of remaining LMO (30-90%). Modeling details are described in the Methods. Initial LMO composition: Ni = 415 ppm[14], Co =115 ppm[14].

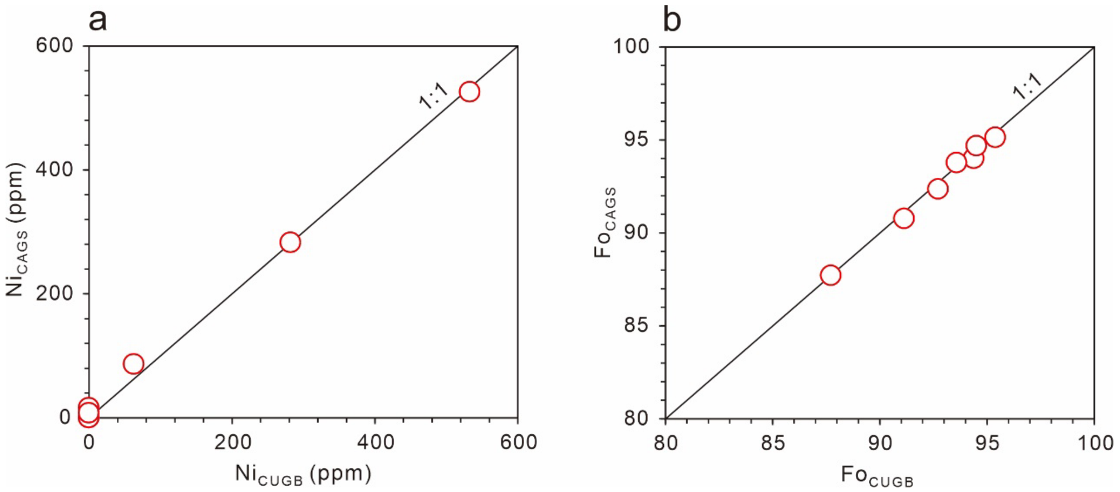

Supplementary Fig. 9. Comparison of Fo values and Ni contents of a random set of olivines by EPMA at China University of Geosciences, Beijing (CUGB) versus at Chinese Academy of Geological Sciences (CAGS).

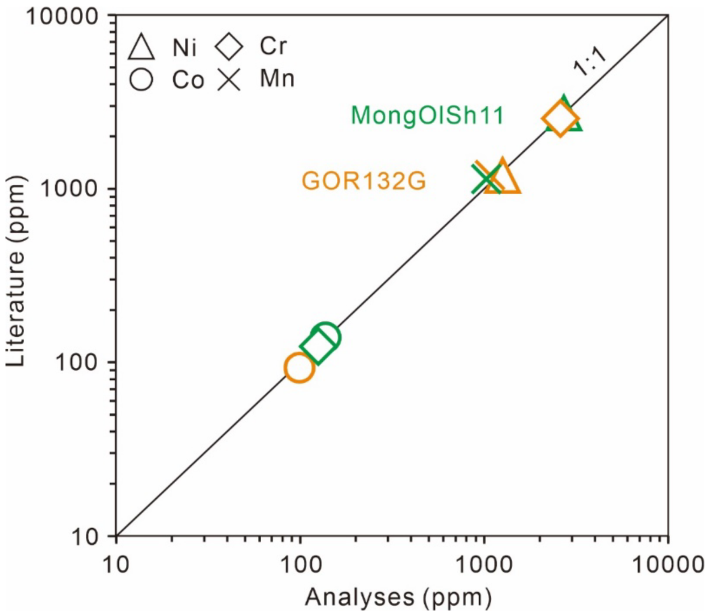

Supplementary Fig. 10. The accuracy of reference materials MongOlSh11-2 and GOR132-G in LA-ICPMS analysis. Data were calculated using ARM-1 as calibration material. The reference values of MongOlSh11-2 and GOR132-G are from ref. 15 and the GeoReM database (http://georem.mpch-mainz.gwdg.de/)[16,17], respectively.

References:

1 Elardo, S. M., Laneuville, M., McCubbin, F. M. & Shearer, C. K. Early crust building enhanced on the Moon's nearside by mantle melting-point depression. *Nature Geoscience* **13**, 339-343 (2020).

2 Ariskin, A. Parental magmas of lunar troctolites: Genetic problems and estimated original compositions. *Geochemistry International* **45**, 413-427 (2007).

3 Longhi, J., Durand, S. R. & Walker, D. The pattern of Ni and Co abundances in lunar olivines. *Geochimica et Cosmochimica Acta* **74**, 784-798 (2010).

4 Prissel, T. C. & Gross, J. On the petrogenesis of lunar troctolites: New insights into cumulate mantle overturn & mantle exposures in impact basins. *Earth and Planetary Science Letters* **551**, 116531 (2020).

5 Prissel, T. C., Parman, S. W. & Head, J. W. Formation of the lunar highlands Mg-suite as told by spinel. *American Mineralogist* **101**, 1624-1635 (2016).

6 Lindstrom, M., Marvin, U. & Mittlefehldt, D. Apollo 15 Mg-and Fe-norites-A redefinition of the Mg-suite differentiation trend. *In: Lunar and Planetary Science Conference, 19th* **19**, 245-254 (1989).

7 Elardo, S. M. & Manosalva, D. F. A. Complexity and ambiguity in the relationships between major lunar crustal lithologies and meteoritic clasts inferred from major and trace element modeling. *Geochimica et Cosmochimica Acta* **354**, 13-26 (2023).

8 Gross, J., Treiman, A. H. & Mercer, C. N. Lunar feldspathic meteorites: Constraints on the geology of the lunar highlands, and the origin of the lunar crust. *Earth and Planetary Science Letters* **388**, 318-328 (2014).

9 Roeder, P. & Emslie, R. Olivine-liquid equilibrium. *Contributions to mineralogy and petrology* **29**, 275-289 (1970).

10 Longhi, J. A new view of lunar ferroan anorthosites: Postmagma ocean petrogenesis. *Journal of Geophysical Research: Planets* **108** (2003).

11 Elardo, S. M., Draper, D. S. & Shearer Jr, C. K. Lunar Magma Ocean crystallization revisited: Bulk composition, early cumulate mineralogy, and the source regions of the highlands Mg-suite. *Geochimica et Cosmochimica Acta* **75**, 3024-3045 (2011).

12 Papike, J., Fowler, G., Adcock, C. & Shearer, C. Systematics of Ni and Co in olivine from planetary melt systems: Lunar mare basalts. *American Mineralogist* **84**, 392-399 (1999).

13 Shearer, C. K. & Papike, J. Early crustal building processes on the moon: Models for the petrogenesis of the magnesian suite. *Geochimica et Cosmochimica Acta* **69**, 3445-3461 (2005).

14 Steenstra, E. S. & van Westrenen, W. Siderophile elements in the lunar mantle. *Encyclopedia of Lunar Science*, 1089-1096 (2023).

15 Wu, S. *et al.* Simultaneous quantification of Forsterite content and minor–trace elements in olivine by LA–ICP–MS and geological applications in Emeishan large Igneous Province. *Minerals* **10**, 634 (2020).

16 Jochum, K. P. & Nohl, U. Reference materials in geochemistry and environmental research and the GeoReM database. *Chemical Geology* **253**, 50-53 (2008).

17 Jochum, K. P. *et al.* GeoReM: a new geochemical database for reference materials and isotopic standards. *Geostandards and Geoanalytical Research* **29**, 333-338 (2005).
