# Supplementary material for: Lunar primitive mantle olivine returned by Chang’e-6
Source: Nat Commun. 2025 Apr 23;16:3759. doi: 10.1038/s41467-025-58820-4 (PMC12019214; doi:10.1038/s41467-025-58820-4)
Supplement: Supplementary file 2 — Description of Additional Supplementary Files [file 41467_2025_58820_MOESM2_ESM.pdf]

Description of Additional Supplementary Files

File name: Supplementary Data 1
Description: Chemical composition of olivine, groundmass, impact glass and spinel in this study.

File name: Supplementary Data 2
Description: Oxygen isotope ratios of olivine and reference materials in this study.

File name: Supplementary Data 3
Description: Ni, Co, Cr and Mn contents of reference materials by LA-ICPMS in this study.
